# Chronic disease burden among Latino farmworkers in California

**DOI:** 10.3389/fpubh.2022.1024083

**Published:** 2022-12-02

**Authors:** Susana L. Matias, Caitlin D. French, Alexander Gomez-Lara, Marc B. Schenker

**Affiliations:** ^1^Nutritional Sciences and Toxicology, University of California, Berkeley, Berkeley, CA, United States; ^2^Public Health Sciences, University of California, Davis, Davis, CA, United States

**Keywords:** farmworkers, agricultural workers, Latino/Hispanic, California, obesity, blood pressure, waist circumference, cholesterol

## Abstract

Farmworkers are an essential workforce to maintain California's extensive agricultural production. However, this mostly Latino, immigrant population is affected by high poverty rates and food insecurity, which increases their risk of chronic diseases. We analyzed clinical and interview data from three studies of Latino farmworkers in California: (1) the Mexican Immigration to California: Agricultural Safety and Acculturation (MICASA) study, (2) the PASOS SALUDABLES pilot intervention (PASOS Pilot), and (3) the PASOS Study, a cluster-randomized, controlled trial (PASOS RCT). We aimed to determine the prevalence of diet-related chronic health outcomes (obesity, elevated waist circumference, high blood pressure, and high total cholesterol) and identify sociodemographic and socioeconomic factors associated with these conditions in this population. A total of 1,300 participants were included in this study (452 from MICASA, 248 from PASOS Pilot, and 600 from PASOS RCT). Obesity prevalence ranged from 29.2 to 54.5% across samples; elevated waist circumference was observed in 29.4–54.0% of participants; high blood pressure was detected in 42.0–45.5% of participants; 23.7–25.8% of participants had high total cholesterol. Age was positively associated with each health outcome, although not for each sample; each additional year in age increased odds by 3–9%, depending on the outcome and sample. Females were at higher risk of obesity (one sample) and elevated waist circumference, but at lower risk of high blood pressure and high total cholesterol. Single, divorced or widowed participants (vs. married/living together) had 35 and 47% reduced odds of obesity and elevated waist circumference, respectively. Each additional year living in the US was associated with 3–6% increased odds of obesity, depending on the sample. Higher household income was associated with a reduction in odds of high total cholesterol up to 76% (one sample). These findings highlight the increased risk of chronic health conditions in Latino farmworkers, in particular for obesity, and among farmworkers who may lack access to health care, which represents a large proportion of this population. Differences in chronic health risks by sex suggest that clinical and public health responses might need to be sex-specific. Expansion of eligibility for supplemental nutrition programs for this low-income population could reduce their disease burden.

## Introduction

An estimated workforce of ~800,000 farmworkers (1) allow California to produce over two-thirds of the fruits and nuts, and over one third of the vegetables, consumed in US households (2). In 2015–2019, this workforce was predominantly Hispanic (96%), was comprised mostly of males (69%), and had an average school level of 8th grade (3). Despite their essential workers classification, farmworkers are poorly compensated and lack access to basic protections. In 2017–2018, 71% of farmworkers reported an annual income of <$30,000 and the majority did not receive assistance from government need-based programs (4). Such low income levels can limit access to adequate food, creating food insecurity (5). Unsurprisingly, food insecurity affects farmworkers at higher rates than the overall US population (6–12), and may increase their risk of several chronic health conditions, such as diabetes (13–15), hypertension (15), hyperlipidemia (14), and overweight/obesity, in particular among females (16–19).

In addition, about 1 in 2 farmworkers in California lacked work authorization, and 42% did not have health insurance, per data collected in 2015–2019; the high cost of health care visits was listed as the most common barrier to accessing health care (3). Despite these socioeconomic risk factors for adverse health, research on farmworkers' health is sparse.

The only representative survey of farmworkers that measured clinical outcomes was conducted in the late 90s in California (20). At that time, more than 70% of farmworkers had overweight or obesity, which was 25% higher than the age-adjusted prevalence in the overall US population in 1999–2000 (21). Furthermore, farmworkers' rates of high blood pressure and high cholesterol were higher among males (27 and 17%, respectively) than females (4% for both conditions) (20). More recently, another study in California suggested an increase in some diet-related health conditions in the farmworker population. Self-reported data from 293 farmworkers in Sonoma County indicated that 94% had overweight or obesity, 15% were ever diagnosed with diabetes, and 26% were ever diagnosed with hypertension (22). Since 1 in 2 farmworkers utilizes some form of US health care, estimates from self-reported data may miss a considerable fraction of this population with undiagnosed and untreated chronic diseases (23).

Lifestyle recommendations for prevention or management of the chronic conditions above include eating a healthy diet and doing exercise. However, socioeconomic and other structural factors influence an individual's ability to follow these recommendations. Growing evidence suggests that these social determinants of health (SDoH) are fundamental causes (or barriers for the management) of a wide range of health outcomes (24). In a study of Latino farmworkers with diabetes in Florida, most farmworkers were knowledgeable about recommended behavioral changes to manage the disease but had difficulty doing so because of the high cost of supplies (e.g., test strips) and limited financial resources to follow a diabetic diet and make other lifestyle changes (25).

To expand the limited research evidence on farmworkers' health, we analyzed clinical and interview data from three studies of Latino farmworkers in California (26–28) to (1) determine the prevalence of diet-related chronic health outcomes, i.e., obesity, elevated waist circumference, high blood pressure (two samples only), and high total cholesterol (two samples only) in this population; and (2) identify sociodemographic and socioeconomic factors (i.e., age, sex, marital status, education, and income) that were independently associated with these conditions among farmworkers, a mostly immigrant, low-income population. Understanding the role of SDoH for specific chronic health outcomes in this population can support evidence-based policy and public health interventions to improve farmworkers' health.

## Methods

### Study design

This study was a secondary data analysis that utilized data collected in three different studies of farmworkers: (1) the Mexican Immigration to California: Agricultural Safety and Acculturation (MICASA) study, a population-based, cohort study of occupational and environmental risks and associated health outcomes, with a two-stage sampling design including random selection of census blocks and door-to-door enumeration (26); (2) the PASOS SALUDABLES pilot intervention (PASOS Pilot), a randomized, controlled, delayed intervention design that allocated farmworker participants to a lifestyle intervention delivered at community health clinics, or to a control group; and (3) the PASOS Study, a cluster-randomized, controlled trial, where clusters (i.e., ranches) were allocated to the intervention (i.e., lifestyle intervention delivered at the ranches) or the control group (PASOS RCT) (28). For MICASA, we used data from the first follow-up interview (exposure measures) and from an ancillary study conducted around the same time (outcome measures). For PASOS Pilot and PASOS RCT, we used baseline data only (i.e., exposure and outcome measured concurrently), because these interventions aimed to change the outcomes reported here. Primary findings from both intervention studies have been previously published (27, 29).

### Study settings

The three studies were conducted in agricultural rich areas in California. The MICASA study was conducted in Mendota, a city in Fresno County in the Central Valley with a high proportion of immigrants and farmworkers residents. The PASOS Pilot study was conducted in two different geographical areas where the study partner (a berry grower company) runs health clinics for its employees: Watsonville, in Northern California, and Oxnard, in Southern California. The PASOS RCT study was also conducted in Oxnard, in partnership with the same berry grower company.

### Participant eligibility

Eligible participants for the MICASA study included males and females aged 18–55 years who self-identified as Mexican or Central American, resided in Mendota at the time of the baseline interview, and whose household included at least one member engaged in farm work (≥45 days in the past year). When possible, both the head of household and spouse were enrolled.

Inclusion criteria for PASOS Pilot included being an employee of the partner grower, ages 18–60 years, body mass index (BMI) 20–38 kg/m^2^, having plans to remain in the area for the next 6 months, being willing to attend 10 weekly education sessions, and being able to read and speak Spanish. Exclusion criteria were: diabetes, pregnancy (or trying to get pregnant) or breastfeeding, taking medications or on therapeutic diets that affect weight, having a medical condition that proscribed physical activity, or having a spouse/partner already in the study. In addition, participants had to carry (worksite, public or private) health insurance, a condition of the study sponsor.

Eligible clusters for PASOS RCT were defined as ranches with ~100 farmworkers. Among those eligible, study ranches were randomly allocated to the intervention group and to the control group. At the individual level, inclusion criteria were being employed by the berry grower, at least 18 years of age, planning to stay in the area for the following 3 months, willing to attend weekly sessions for 6–12 weeks, and able to read and speak Spanish. Exclusion criteria included: unable to communicate in Spanish, pregnant, planning a pregnancy or breastfeeding, unable to do moderate physical exercise, taking medicine for high blood pressure or heart conditions, having bone or joint problems, experiencing loss of consciousness or falls due to dizziness, or having developed chest pain within the last month, taking medications that affect weight or following therapeutic diets, previous diabetes diagnosis or HbA1c ≥ 6.5% at screening, having a spouse/cohabitant already enrolled in the study, and having previously participated in the employer's lifestyle intervention.

### Data collection

The MICASA follow-up data were collected from November 2008 to February 2010, and the anthropometric and clinical data were collected between February 2009 and February 2010, as part of an ancillary study to assess lung function. PASOS Pilot data was collected between April 2010 and January 2011. Anthropometric measurements were collected, and an interviewer-administered questionnaire was given in Spanish by a research assistant at a worksite clinic. PASOS RCT baseline data was collected between August 2015 and August 2017. All interview data and clinical measurements were collected at an employer-run health clinic by two trained bilingual research assistants.

In each study, the interviewer-administered questionnaire included questions on sociodemographic characteristics, along with other questions (e.g., occupational and environmental risk factors, injuries, etc.). Specifically, participants were asked their date of birth, sex, the number of years of schooling they had completed, marital status, family's total income in the previous year, country of birth, and the number of years they have been living in the US, using similar questions in all three studies. Study questionnaires were translated into Spanish and then back translated into English by bilingual individuals. All participants provided written informed consent. The study protocols were approved by the University of California, Davis Institutional Review Board.

### Study outcomes

Anthropometric measurements were collected using standardized protocols (e.g., wearing light clothing, no shoes, etc.), and equipment calibration procedures were implemented. Two measures were taken and averaged; a third measure was recorded when the first two measurements were not within a pre-determined maximum difference.

To measure weight (lb) and height (in) in the MICASA study, a Seca 703 physician scale with attached stadiometer (SECA, Chino, CA) was used. In the other two studies, weight (kg) was measured using an EatSmart Precision Digital Bathroom Scale (Health Tools, LLC., Wyckoff, NJ). Standing height (cm) was measured with a Seca 213 mobile stadiometer (SECA, Chino, CA). BMI was calculated as weight in kg divided by height in meters squared (kg/m^2^). Obesity status was defined as BMI ≥30 kg/m^2^ (30). Participants categorized as underweight (BMI <18.5 kg/m^2^; *n* = 2) were excluded from the obesity variable (therefore not included in any analysis involving this variable).

Waist circumference (cm) was measured against the skin at the natural waist with a Medline Disposable Paper Tape Measure (Medline Industries, Inc., Lathrop, CA) in PASOS Pilot and with a Gulick II tape measure, Model 67020 (Country Technology Inc., Gays Mills, WI) in MICASA and PASOS RCT. Waist circumference values >40 inches (for males) or >35 inches (for females) were categorized as elevated (31).

Blood pressure (mm Hg) was measured with an automated device that employs standardized Doppler procedures; systolic and diastolic blood pressure measures were taken in duplicate and the values were averaged. High blood pressure was defined as systolic blood pressure ≥130 mm Hg or diastolic blood pressure ≥80 mm Hg (32).

Total cholesterol (mg/dL) was assessed using the Cholestech LDX^®^ System (Cholestech Corporation, Hayward, CA). High total cholesterol was defined as ≥200 mg/dL (33).

### Statistical analysis

All MICASA data were analyzed considering the study sampling design (26), i.e., including census blocks as clusters, and census tracts as strata. PASOS RCT data was also analyzed considering its cluster sampling design, by including the ranches as clusters in all analyses.

Descriptive statistics to characterize the study sample and health outcomes consisted of frequency distributions with standard errors for categorical data, and means and standard errors for continuous data. We used standard errors to calculate 95% confidence intervals (CI) for visual comparison among samples.

This analysis was focused on quantifying the *total* association of the study exposures (i.e., sociodemographic or socioeconomic factors) with study outcomes, without considering other more proximal variables potentially in the causal path, such as dietary intake (34). Thus, unadjusted and adjusted logistic regression models were used to identify the sociodemographic/socioeconomic factors associated with each outcome. Sociodemographic/socioeconomic factors associated with a health outcome at p <0.20 were included in the multivariate (adjusted) model, to determine their independent association with that specific outcome. Unadjusted odds ratios (OR) and adjusted odds ratios (AOR) were estimated, along with their corresponding 95% CI.

An available case analysis approach was followed, i.e., no imputation of missing data was done. All hypothesis testing was two-sided, using a 5% level of significance. All data were analyzed using SAS version 9.4 (SAS Institute, Inc., Cary, NC).

## Results

The MICASA cohort consisted of 843 participants enrolled at baseline; 640 of them completed the first follow up assessment and 453 completed the ancillary study (when clinical measurements were taken). The MICASA sample for this analysis included *n* = 452 participants who completed both assessments. In total, 254 farmworkers were enrolled in the PASOS Pilot; after excluding participants who were not eligible at baseline (i.e., who had diabetes, determined as fasting blood sugar values ≥126 mg/dL), the analytic sample for the PASOS Pilot study was *n* = 248. A total of 615 farmworkers were enrolled in the PASOS RCT trial, but 15 of them were deemed not eligible due to diabetes at baseline (i.e., HbA1c values ≥ 6.5%). Thus, *n* = 600 participants were retained for this analysis. Adding up the three samples resulted in 1,300 participants.

Sociodemographic characteristics of the three samples are described in Table 1. Briefly, participants were relatively young, in particular in the PASOS Pilot and PASOS RCT studies, mostly married, with low education levels. Most participants were immigrants from Mexico, and the majority of PASOS Pilot and PASOS RCT participants reported Spanish as their primary language, while some participants (12–22%) indicated an Indigenous language (primary language was not assessed in MICASA).

**Table 1:** Sociodemographic characteristics of Latino farmworkers in California (*n* = 1,300)*^a^*.

	**MICASA (*n* = 452)**	**PASOS Pilot (*n* = 248)**	**PASOS RCT (*n* = 600)**
	***n*** **(%)**		
Age, y [Mean (SE)]	40.9 (1.1)	32.3 (0.5)	33.9 (0.5)
Sex			
Male	185 (40.9)	69 (27.8)	315 (52.8)
Female	267 (59.1)	179 (72.2)	282 (47.2)
Education			
No school	26 (5.8)	6 (2.5)	27 (4.5)
Primary education or less	267 (59.2)	124 (51.9)	279 (46.7)
More than primary	158 (35.0)	109 (45.6)	292 (48.8)
education			
Income, USD			
≤ $10,000	54 (12.6)	52 (21.8)	¥
$10,001–$20,000	128 (30.0)	86 (36.1)	¥
$20,001–$30,000	134 (31.4)	62 (26.1)	¥
>$30,000	111 (26.0)	38 (16.0)	¥
Marital status			
Married/Living together	427 (94.5)	190 (76.9)	399 (66.8)
Single/Divorced/Widow	25 (5.5)	57 (23.1)	198 (33.1)
Primary language			
Spanish	–	218 (87.9)	449 (74.8)
Indigenous	–	29 (11.7)	133 (22.2)
Other	–	1 (0.4)	18 (3.0)
Country of birth			
US	13 (2.9)	1 (0.4)	1 (0.2)
Mexico	303 (67.0)	246 (99.2)	580 (96.7)
Central America	136 (30.1)	1 (0.4)	19 (3.2)
Years in the US [Mean (SE)]	18.2 (0.7)	11.4 (0.4)	¥

a *n* (%) are presented, unless otherwise indicated.

–Indicates that variable was not measured in that study.

¥ Indicates that data for that variable was not available for this analysis.

MICASA, Mexican Immigration to California: Agricultural Safety and Acculturation study; RCT, randomized controlled trial; SE, Standard Error; USD, US dollars.

Table 2 shows the clinical data available for each study. Mean BMI values in the three samples were between 28.0 and 31.3 kg/m^2^, which fall in the overweight or obesity category. Mean waist circumference ranged from 88.8 to 97.0 cm across the samples. Mean systolic blood pressure was higher in the MICASA compared to the PASOS RCT participants (127.4 vs. 120.2 mmHg); mean diastolic blood pressure ranged from 73.6 to 76.9 mmHg. Mean total cholesterol values ranged from 174 to 175 mg/dL.

**Table 2:** Clinical indicators and health outcomes in Latino farmworkers in California (*n* = 1,300).

	**MICASA (*n* = 452)**	**PASOS Pilot (*n* = 248)**	**PASOS RCT (*n* = 600)**
	**Mean (95% CI)**		
Body mass index, kg/m^2^	31.3 (30.7, 31.9)	28.0 (27.5, 28.5)	28.4 (27.9, 28.8)
Waist circumference, cm	97.0 (95.8, 98.1)	88.8 (87.5, 90.1)	90.8 (89.9, 91.7)
Systolic blood pressure, mm Hg	127.4 (125.5, 129.3)	–	120.2 (118.2, 122.2)
Diastolic blood pressure, mm Hg	73.6 (72.7, 74.6)	–	76.9 (75.8, 78.0)
Total cholesterol, mg/dL	–	173.6 (167.7, 179.5)	174.6 (170.0, 179.1)
	**Percent (95% CI)**		
Weight status			
Underweight	0.2 (−0.2, 0.7)	0 (N/A)	0.2 (−0.2, 0.5)
Normal weight	8.2 (5.7, 10.8)	27.4 (21.9, 33.0)	22.3 (19.4, 25.2)
Overweight	37.2 (33.2, 41.1)	39.5 (33.4, 45.6)	48.3 (45.0, 51.7)
Obesity class 1	35.2 (31.6, 38.8)	28.2 (22.6, 33.8)	21.2 (17.7, 24.6)
Obesity class 2	12.7 (9.5, 15.9)	4.8 (2.2, 7.5)	6.2 (4.4, 7.9)
Obesity class 3	6.5 (4.4, 8.5)	0 (N/A)	1.8 (0.9, 2.7)
Blood pressure status			
Normal	36.4 (32.1, 40.6)	–	45.2 (38.3, 52.1)
Elevated	18.2 (14.8, 21.5)	–	12.8 (9.5, 16.2)
Hypertension stage 1	30.2 (25.4, 34.9)	–	37.0 (33.2, 40.8)
Hypertension stage 2	15.3 (11.5, 19.1)	–	5.0 (3.5, 6.5)
Study outcomes			
Obesity	54.5 (49.3, 59.6)	33.1 (27.2, 38.9)	29.2 (25.1, 33.3)
Elevated waist circumference	54.0 (48.3, 59.7)	38.3 (32.2, 44.4)	29.4 (25.0, 33.8)
High blood pressure	45.5 (39.9, 51.0)	–	42.0 (37.1, 46.9)
High total cholesterol	–	25.8 (20.3, 31.3)	23.7 (18.7, 28.7)

MICASA, Mexican Immigration to California: Agricultural Safety and Acculturation study; RCT, randomized controlled trial; CI, Confidence Interval.

–Indicates that variable was not measured in that study.

**Figure 1:**
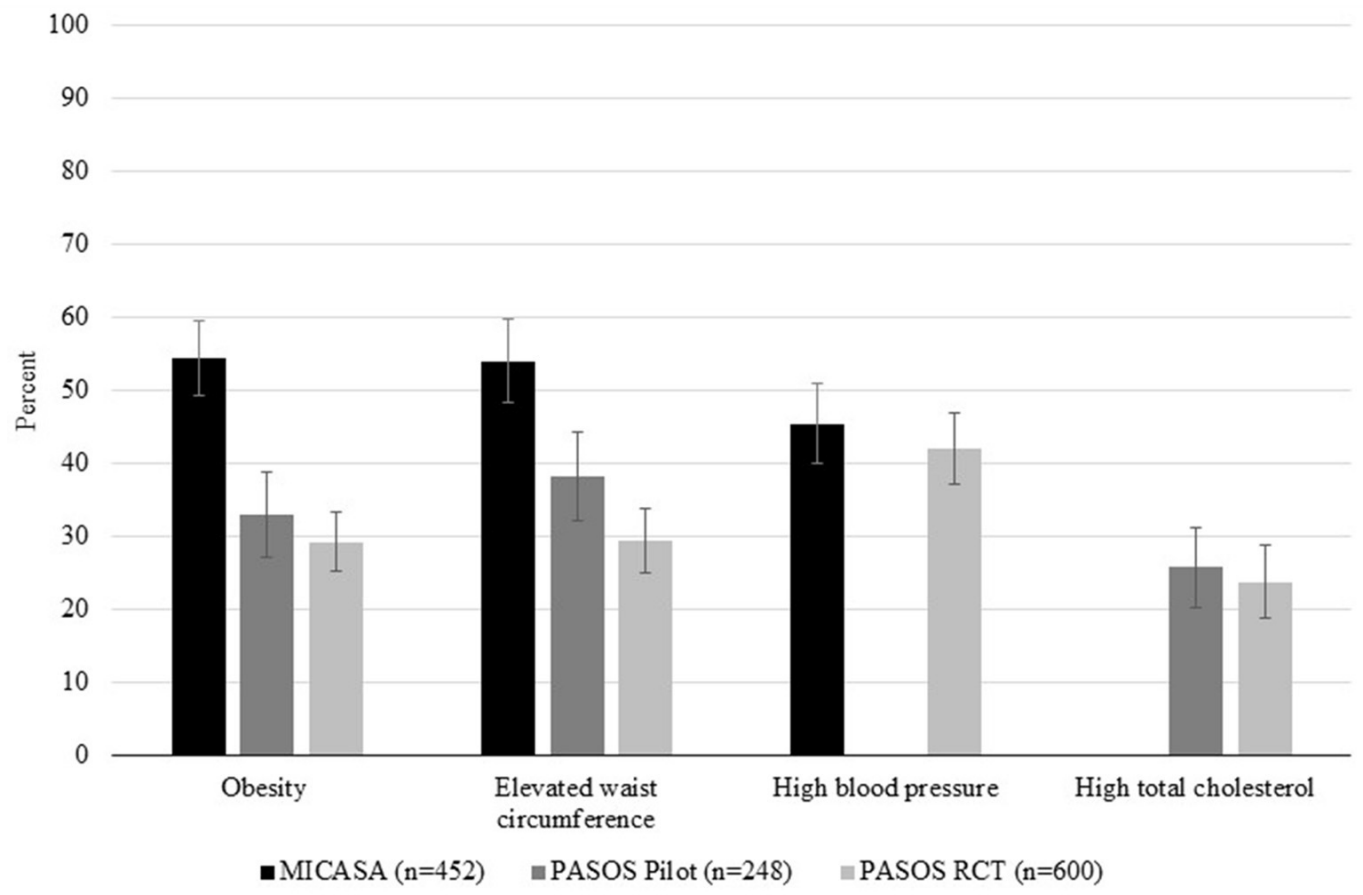
Health outcomes in Latino farmworkers in California (*n* = 1,300). Percent of participants with obesity, elevated waist circumference, high blood pressure and high total cholesterol in each sample. MICASA data is represented by black bars, PASOS Pilot data is represented by dark gray bars, and PASOS RCT data is represented by light gray bars. Bars represent 95% confidence intervals. Empty spaces indicate that an outcome was not measured in that sample (i.e., blood pressure in PASOS Pilot and total cholesterol in MICASA).

Prevalence of obesity ranged from 29.2% in the PASOS RCT to 54.5% in the MICASA sample (Figure 1). Elevated waist circumference was observed in 29.4–54.0% of participants across samples. High blood pressure was detected in 42.0–45.5% of participants. The proportion of participants with high total cholesterol ranged from 23.7 to 25.8%.

Tables 3–**6** show estimates of the association (odds ratios) and 95% CI based on unadjusted (individual sociodemographic/socioeconomic factors) and adjusted analyses (including factors associated with each outcome in unadjusted analysis) for each health outcome in each sample. For obesity (Table 3), females in the MICASA study had 63% higher odds of having obesity than males, after adjusting for all other sociodemographic variables associated with this outcome (*p* = 0.015). PASOS RCT participants who were single, divorced or widowed had 35% reduced odds of obesity vs. those that were married or living together (*p* = 0.025). Also, each additional year in age was associated with a 3 and 7% increased odds of obesity among PASOS Pilot (*p* = 0.002) and PASOS RCT (*p* = 0.033) participants, respectively. In the two samples for which years living in the US was analyzed, every additional year in the US was associated with 3 and 6% higher odds of obesity in MICASA (*p* = 0.047) and PASOS Pilot (*p* = 0.036), respectively.

**Table 3:** Unadjusted and adjusted associations between sociodemographic exposures and obesity among Latino farmworkers in California.

	**MICASA**		**PASOS pilot**		**PASOS RCT**	
	**OR (95% CI)**	**AOR*^a^* (95% CI)**	**OR (95% CI)**	**AOR*^a^* (95% CI)**	**OR (95% CI)**	**AOR*^a^* (95% CI)**
Age, y	1.02 (1.00, 1.04)	1.01 (0.98, 1.03)	1.10 (1.06, 1.15)	**1.07 (1.03, 1.12)**	1.04 (1.01, 1.06)	**1.03 (1.00, 1.05)**
Sex*^b^*						
Female	1.38 (0.95, 2.01)	**1.63 (1.10, 2.41)**	0.98 (0.55, 1.77)	§	1.74 (0.97, 3.13)	1.59 (0.92, 2.74)
Education*^c^*						
>Primary education	0.78 (0.49, 1.24)	§	1.03 (0.60, 1.78)	§	0.57 (0.38, 0.85)	0.69 (0.46, 1.05)
Income, USD*^d^*						
$10,001–$20,000	1.05 (0.47, 2.34)	§	0.71 (0.34, 1.48)	§		
$20,001–$30,000	1.24 (0.68, 2.27)	§	0.89 (0.41, 1.92)	§		
>$30,000	0.96 (0.49, 1.88)	§	1.13 (0.48, 2.68)	§		
Marital status*^e^*						
Single/Divorced/Widow	0.76 (0.31, 1.86)	§	0.73 (0.38, 1.41)	§	0.65 (0.45, 0.93)	**0.65 (0.45, 0.94)**
Years in the US	1.03 (1.00, 1.06)	**1.03 (1.00, 1.07)**	1.12 (1.07, 1.17)	**1.06 (1.00, 1.13)**		

a Adjusted for all other sociodemographic variables associated with the outcome at *p* < 0.20 in unadjusted analyses. Significant associations (*p* < 0.05) are indicated in bold text.

b Reference: Male.

c Reference: Less than primary education.

d Reference: ≤ $10,000.

e Reference: Married/Living together.

§Variable not included in adjusted model because unadjusted association was not significant at the *p* < 0.20 level.

AOR, Adjusted Odds Ratio; CI, Confidence Interval; MICASA, Mexican Immigration to California: Agricultural Safety and Acculturation study; OR, Odds Ratio; RCT, randomized controlled trial; USD, US dollars.

For waist circumference (Table 4), after adjustment as described above, females had more than two-, three-, and six-fold higher odds of having elevated waist circumference than males, in the PASOS Pilot (*p* = 0.008), MICASA (*p* < 0.0001), and PASOS RCT (*p* < 0.0001), respectively. Every additional year in age was associated with a 5% (MICASA, *p* = 0.001; PASOS RCT, *p* = 0.005) and 9% increased odds (PASOS Pilot, *p* < 0.001) of elevated waist circumference. A negative association was found with being single, divorced or widowed (compared to being married or living together) in the PASOS RCT (47% reduced odds, *p* = 0.006), but not in the MICASA (*p* = 0.353) or PASOS Pilot (*p* = 0.632) samples.

**Table 4:** Unadjusted and adjusted associations between sociodemographic exposures and elevated waist circumference among Latino farmworkers in California.

	**MICASA**		**PASOS pilot**		**PASOS RCT**	
	**OR (95% CI)**	**AOR*^a^* (95% CI)**	**OR (95% CI)**	**AOR*^a^* (95% CI)**	**OR (95% CI)**	**AOR*^a^* (95% CI)**
Age, y	1.05 (1.03, 1.07)	**1.05 (1.02, 1.07)**	1.10 (1.06, 1.15)	**1.09 (1.04, 1.14)**	1.06 (1.02, 1.09)	**1.05 (1.02, 1.09)**
Sex*^b^*						
Female	2.75 (2.02, 3.73)	**3.73 (2.52, 5.50)**	2.91 (1.53, 5.54)	**2.49 (1.27, 4.90)**	6.61 (4.50, 9.73)	**6.63 (4.73, 9.28)**
Education*^c^*						
>Primary education	0.74 (0.51, 1.09)	0.79 (0.55, 1.13)	0.84 (0.50, 1.42)	§	0.57 (0.38, 0.86)	0.80 (0.51, 1.24)
Income, US*^d^*						
$10,001–$20,000	1.05 (0.55, 2.01)	§	0.66 (0.32, 1.34)	§		
$20,001–$30,000	1.13 (0.68, 1.89)	§	1.12 (0.53, 2.36)	§		
>$30,000	1.02 (0.60, 1.71)	§	0.71 (0.30, 1.69)	§		
Marital status*^e^*						
Single/Divorced/ Widow	2.28 (0.98, 5.35)	1.46 (0.65, 3.31)	0.49 (0.26, 0.95)	0.63 (0.31, 1.30)	0.62 (0.42, 0.90)	**0.53 (0.35, 0.80)**
Years in the US	1.03 (1.00, 1.06)	1.02 (0.99, 1.06)	1.09 (1.04, 1.14)	1.02 (0.96, 1.08)		

a Adjusted for all other sociodemographic variables associated with the outcome at *p* < 0.20 in unadjusted analyses. Significant associations (*p* < 0.05) are indicated in bold text.

b Reference: Male.

c Reference: Less than primary education.

d Reference: ≤ $10,000.

e Reference: Married/Living together.

§Variable not included in adjusted model because unadjusted association was not significant at the p < 0.20 level.

AOR, Adjusted Odds Ratio; CI, Confidence Interval; MICASA, Mexican Immigration to California: Agricultural Safety and Acculturation study; OR, Odds Ratio; RCT, randomized controlled trial; USD, US dollars.

Blood pressure and cholesterol were measured in two studies only (Table 5). In adjusted analyses, females had 55 and 74% reduced odds of having high blood pressure compared to males in the MICASA (*p* < 0.0001) and PASOS RCT (*p* < 0.0001) samples, respectively. In addition, every additional year in age was associated with 5% higher odds of high blood pressure in both MICASA (*p* < 0.001) and PASOS RCT (*p* = 0.001).

**Table 5:** Unadjusted and adjusted associations between sociodemographic exposures and high blood pressure among Latino farmworkers in California.

	**MICASA**		**PASOS RCT**	
	**OR (95% CI)**	**AOR*^a^* (95% CI)**	**OR (95% CI)**	**AOR*^a^* (95% CI)**
Age, y	1.06 (1.04, 1.08)	**1.05 (1.03, 1.07)**	1.04 (1.01, 1.06)	**1.05 (1.02, 1.08)**
Sex*^b^*				
Female	0.40 (0.29, 0.55)	**0.45 (0.32, 0.65)**	0.31 (0.22, 0.44)	**0.26 (0.17, 0.40)**
Education*^c^*				
>Primary education	0.86 (0.59, 1.23)	§	0.96 (0.69, 1.33)	§
Income, USD*^d^*				
$10,001–$20,000	1.49 (0.71, 3.07)	§		
$20,001–$30,000	1.41 (0.70, 2.86)	§		
>$30,000	1.20 (0.60, 2.38)	§		
Marital status*^e^*				
Single/Divorced/ Widow	0.79 (0.39, 1.59)	§	0.89 (0.63, 1.25)	§
Years in the US	1.05 (1.03, 1.07)	1.01 (0.99, 1.04)		

a Adjusted for all other sociodemographic variables associated with the outcome at *p* < 0.20 in unadjusted analyses. Significant associations (*p* < 0.05) are indicated in bold text.

b Reference: Male.

c Reference: Less than primary education.

d Reference: ≤ $10,000.

e Reference: Married/Living together.

§Variable not included in adjusted model because unadjusted association was not significant at the p < 0.20 level.

AOR, Adjusted Odds Ratio; CI, Confidence Interval; MICASA, Mexican Immigration to California: Agricultural Safety and Acculturation study; OR, Odds Ratio; RCT, randomized controlled trial; USD, US dollars.

For cholesterol (Table 6), adjusted analyses revealed that females had 60 and 92% reduced odds of having high total cholesterol, compared to males, in the PASOS RCT (*p* < 0.0001) and PASOS Pilot (*p* = 0.001), respectively. Participants with household incomes between $10,001–$20,000 USD and $20,001–$30,000 USD had 76 and 69% reduced odds of high total cholesterol (respectively) vs. those whose household income was below $10,000 USD in the PASOS Pilot (*p* = 0.019). Every additional year in age was associated with 5% increased odds of high total cholesterol in the PASOS RCT (*p* < 0.0001), while age was not significantly associated with high cholesterol in the PASOS Pilot (*p* = 0.587).

**Table 6:** Unadjusted and adjusted associations between sociodemographic exposures and high cholesterol among Latino farmworkers in California.

	**PASOS pilot**		**PASOS RCT**	
	**OR (95% CI)**	**AOR*^a^* (95% CI)**	**OR (95% CI)**	**AOR*^a^* (95% CI)**
Age, y	0.96 (0.93, 1.00)	0.98 (0.92, 1.05)	1.05 (1.03, 1.07)	**1.05 (1.04, 1.07)**
Sex*^b^*				
Female	0.08 (0.04, 0.16)	**0.08 (0.04, 0.17)**	0.45 (0.30, 0.67)	**0.40 (0.26, 0.61)**
Education*^c^*				
>Primary education	2.36 (1.31, 4.28)	1.97 (0.91, 4.24)	0.95 (0.68, 1.32)	§
Income, USD*^d^*				
$10,001–$20,000	0.37 (0.17, 0.80)	**0.24 (0.09, 0.64)**		
$20,001–$30,000	0.47 (0.21, 1.06)	**0.31 (0.11, 0.87)**		
>$30,000	0.74 (0.31, 1.79)	0.67 (0.22, 2.11)		
Marital status*^e^*				
Single/Divorced/ Widow	1.06 (0.54, 2.07)	§	1.12 (0.61, 2.06)	§
Years in the US	0.97 (0.92, 1.02)	1.02 (0.94, 1.11)		

a Adjusted for all other sociodemographic variables associated with the outcome at *p* < 0.20 in unadjusted analyses. Significant associations (*p* < 0.05) are indicated in bold text.

b Reference: Male.

c Reference: Less than primary education.

d Reference: ≤ $10,000.

e Reference: Married/Living together.

§Variable not included in adjusted model because unadjusted association was not significant at the p < 0.20 level.

AOR, Adjusted Odds Ratio; CI, Confidence Interval; MICASA, Mexican Immigration to California: Agricultural Safety and Acculturation study; OR, Odds Ratio; USD, US dollars.

## Discussion

Findings from this study confirm the high chronic disease burden previously reported in the farmworker population. In a representative sample of farmworker households in Mendota, California, 55% had obesity, 54% had elevated waist circumference, and 46% had high blood pressure. In the other two samples of farmworkers that excluded certain medical conditions (i.e., diabetes, and class 3 obesity), 29–33% of farmworkers had obesity, 29–38% had elevated waist circumference, 24–26% had high total cholesterol, depending on the sample, and 42% had high blood pressure (in one study). The PASOS Pilot and PASOS RCT samples likely represented a healthier group among farmworkers, and possibly among Latinos and the overall US population, due to the exclusion of the health conditions stated above, and the inclusion criterion of having health insurance.

### Obesity

Because the MICASA study included a population-based sample of farmworkers, comparison of estimates from this cohort with those from the broader Latino and overall US population is more suitable. The obesity prevalence observed in the MICASA cohort (55%) was higher than the 2009–2010 age-adjusted prevalence among all US adults (36%) (35) and the 2007–2010 age-adjusted prevalence among Mexican-Americans (35% in males and 44% in females) (36), a comparable group for the predominantly Mexican-born farmworker population in California. For severe obesity (class 3), the prevalence in the MICASA sample (6.5%) was comparable to the age-adjusted prevalence (6.4%) for all US adults in 2009–2010 (37). Thus, farmworkers may carry a higher burden of obesity-related medical needs than other populations in the US.

Comparing to other Latino farmworker populations, the obesity prevalence in MICASA was higher than that in the only representative survey of agricultural workers in California (38% among females and 29% among males), conducted about 10 years earlier (38), and those reported for Latino farmworkers in Michigan from clinical examinations collected in 2002–2004 (40%) (39) and in Oregon from clinical medical records in 2004–2012 (23%) (40). These comparisons suggest geographic variations and a potentially rising prevalence of obesity among farmworker populations, although more research in population-based samples is needed to confirm these trends.

Sociodemographic predictors of obesity in the studied samples included age, sex, marital status, and years in the US, although statistical significance was not consistent across samples. Nevertheless, the increased risk of obesity among MICASA females vs. males was consistent with previous findings in farmworkers in California (38) and Michigan (39), and in the overall Latino population in the US (41). Interestingly, this association was not significant in the other two samples including likely healthier farmworkers with health insurance. This finding suggests that future intervention efforts aimed at promoting metabolic health may need to focus on female farmworkers, in particular, in the context of limited access to health care. Age was positively associated with obesity in the PASOS Pilot and PASOS RCT samples (but not in the MICASA study), with each additional year in age resulting in 3–7% higher odds of obesity. This association has been previously reported in a large sample of Latino vineyard and winery farmworkers in Oregon, where those aged 45–64 had 85% increased odds of having obesity compared to farmworkers who were 18–44 years old (40).

A trend toward a negative association with being single, divorced or widowed was consistently present in all three samples, but it only reached significance in the PASOS RCT sample, likely due to the small numbers of farmworkers in that category in the other two samples. This association is consistent with findings from the farmworker study in Oregon, where male and female farmworkers who were married or living together had higher odds of obesity (OR = 1.72, 95% CI: 1.35, 2.21, and OR = 1.57, 95% CI: 1.05, 2.37, respectively) (40).

The association between acculturation and poorer dietary and health related behaviors has been previously established for Mexican Americans (42) and Latinos in general (43–46). In this study, we used years living in the US as a proxy for acculturation and found that each additional year in the US was associated with 3 or 6% increased risk of obesity in the two studies that assessed it. Our results are consistent with those observed among other Latino farmworkers in Oregon, where living in the US for longer (i.e., 10 y or more) was associated with higher odds of obesity for male (OR = 2.08, 95% CI: 1.57–2.76) and female farmworkers (OR = 1.81, 95% CI: 1.07–3.06) (40). This finding suggests that even among farmworkers, a very low acculturated population, small changes in acculturation, such as an extra year in the US, carry a negative, modest but significant, effect on health outcomes. However, the lack of association with the other health outcomes studied here, discussed below, highlights the need for further research in this area.

### Elevated waist circumference

The measurement of waist circumference is recommended as an independent and complementary measure to BMI to better evaluate or manage the cardiometabolic risk associated with increased adiposity (47). In this analysis, 54% of farmworkers in the MICASA sample had elevated waist circumference, similar to the prevalence of abdominal obesity (54.5%, based on the same definition) in the overall US population in 2011–2012 (48), and close to the prevalence of central obesity (57.0%) in the overall Hispanic adult population in 2013–2014, even though the latter outcome was measured differently, i.e., using sagittal abdominal diameter (41).

Similar to what we observed for obesity, significant predictors of elevated waist circumference included age, sex, and marital status. However, the association between years in the US and overall obesity did not translate into a link with elevated waist circumference, suggesting that changes in body composition other than central adiposity may partially explain the association with obesity in this population. Further, in contrast to the inconsistent association between sex and obesity across the samples, a striking finding of this study is the consistent and strong association of elevated waist circumference with female sex. This is concerning given the reported association of this outcome with chronic conditions like diabetes (49), hypertension (50) and liver disease (51), and with all-cause, cardiovascular, and cancer mortality (52). Since increasing parity has been associated with greater waist circumference in females (53, 54), considering this variable in future studies with female farmworkers would be relevant.

### High blood pressure

High blood pressure or hypertension is a major risk factor for cardiovascular disease (55), the leading cause of death in the US (56). Of particular concern was the high prevalence we observed in this study (42.0–45.5%, depending on the sample). These estimates were much higher than the prevalence of hypertension found in the general Latino population around the same time period (~26%) (57, 58) and that previously reported among farmworkers (22, 38). However, in those studies, the definition of hypertension was based on previous guidelines, i.e., higher cut-offs for SBP and DBP, which may partially account for the discrepancy in estimates. Further, the Moore et al. study in farmworkers defined hypertension based on self-reported diagnosis, which likely underestimated the actual prevalence in that sample since <1 in 3 participants had health insurance (22). Despite these limitations in the ability to compare across studies, the finding that close to half of farmworkers in the studied samples had blood pressure readings consistent with hypertension represents a significant public health problem.

Similarly to the obesity-related outcomes (22), increasing age was linked to a 5% increase per year in odds of high blood pressure in both samples where this outcome was assessed. This association with age was also found in the HCHS/SOL (58), and among Latino vineyard and winery farmworkers in Oregon (40). However, we did not detect an association with time living in the US, as was found in the Oregon study, where living in the United States for ≥10 years was associated with higher odds of hypertension in males (OR = 1.89, 95% CI: 1.36–2.65) (40). In contrast to our findings on obesity and waist circumference, female farmworkers had significantly lower odds of having high blood pressure than did males. This sex difference in the prevalence of hypertension has been well-established in the overall population (59). The lower hypertension prevalence among females is evident until middle age or menopause, and may relate to differences in vascular physiology (60), the immune system response to hypertension (61), and/or sex hormones (62), among other potential mechanisms.

### High total cholesterol

The proportions of farmworkers with high total cholesterol (24 and 26%, depending on the sample) were lower than that reported for Hispanic males (46.2%) and females (43.4%) in 2012 (63), and were comparable to the prevalence of hypercholesteremia (total cholesterol ≥200 mg/dL) reported among farmworkers in Oregon (21.6%) (40). This outcome was assessed only in the two samples that excluded other medical conditions and required health insurance, which may represent a healthier group in general. Similarity with the estimate from the Oregon study may relate to potential higher access to health care in that population, as study data was obtained from medical records. Blood cholesterol was also assessed in the California-wide survey of agricultural workers conducted in 1999, but the determination of high cholesterol was based on a higher cut-off (>240 mg/dL) (20), limiting comparability of findings. Future surveys assessing cholesterol or other lipid markers of cardiovascular health (e.g., low-density lipoproteins or LDL) in the general, mostly uninsured, farmworker population could provide insight into the actual CVD risk in this population.

The present study identified sex, age, and income as potentially important sociodemographic/-economic determinants of high total cholesterol among Latino farmworkers. The association with sex was consistent, with females having significantly lower odds than males in both samples. This difference is in agreement with findings from the Villarejo et al. study, despite differences in outcome definition (20). In addition, each additional year in age was associated with 5% increased odds of high total cholesterol in the PASOS RCT, which is consistent with findings among Latino farmworkers in Oregon, where older farmworkers (45–64 y) showed higher odds of hypercholesterolemia compared to those 18–44 y (OR = 2.53, 95% CI: 1.96, 3.28 for males and OR = 3.77, 95% CI: 2.07, 6.86 for females) (40). Finally, higher income was associated with lower odds of high cholesterol in the PASOS Pilot sample; the non-significant result in the highest income category (>$30,000) was likely due to the small number of participants (*n* = 38). Although blood cholesterol level is not a direct reflection of the amount of cholesterol in the diet, it is influenced by the mix of fats and carbohydrates consumed; these, in combination with a low intake of fiber and polyunsaturated fatty acids (PUFA) can increase risk of coronary heart disease (64). Among the barriers for a healthy diet among adults, lower income has been consistently associated with lower consumption of fruits and vegetables (65), which are good sources of fiber, while economic subsidies have increased consumption (66, 67). Moreover, fast-food restaurants and energy-dense foods are more available in lower-income and minority neighborhoods across the US (68), which likely increases consumption due to cheaper prices, compared to healthier food options. Thus, this study finding provides a glimpse of the positive health effects that increasing farmworker wages and improving availability of fresh produce in underserved neighborhoods may have.

Farmworker households may face multiple, interacting barriers to achieving optimal health and wellbeing. Living in poverty, as many farmworkers do, is a major barrier to healthy food access, as it forces one to make difficult choices between meeting other basic needs such as housing costs or medical care and food. Language and low education are barriers to accessing medical care, as individuals with limited English proficiency in California are less likely to have access to preventative care (69). Although most farmworkers speak Spanish, there is a small proportion who speak different indigenous languages (3), making the language barrier a hard one to address. While we had information on primary language for two of the study samples, lack of enough variation prevented us from any further analysis. This highlights the need for research to expand its reach to include this subgroup.

This study has several limitations, such as the inclusion of a limited number of sociodemographic and socioeconomic exposures, or SDoH. Future studies would benefit from including other important factors for this population, such as access to health care and migration patterns. Another limitation was the use of cross-sectional data, which limited our ability to address directional causality in the association between exposures and outcomes. In addition, assessment of blood pressure, although in duplicate, was done in a single visit, and thus did not constitute a medical determination of hypertension, which requires repeat measurements over time showing elevated blood pressure. Also, while this study included samples from three different studies with farmworkers, because each study had a different study design, we were unable to carry out a pooled analysis. Finally, although this study included three samples from different regions in California, one of which was representative of the farmworker population in one region, the study population is not a representative sample of all Latino farmworkers in the state, in particular because of the inclusion and exclusion criteria used in two of the study samples. Therefore, findings may not be generalizable to other farmworker populations. These limitations point to the need for a comprehensive statewide survey study to estimate current chronic disease burden among farmworkers and identify SDoH to guide evidence-based policy changes.

Nevertheless, the study findings contribute to understanding the role of more distal determinants of the chronic disease burden in this immigrant, underserved, low-income population, and support the refocusing of public health and clinical efforts in agricultural areas to work at multiple levels to address prevention and treatment for these chronic conditions in farmworkers. One interesting approach is the Food as Medicine interventions, such as provision of medically tailored meals, food pharmacies and produce prescriptions. These interventions have reduced BMI (70) and blood pressure (71, 72) in clinical patients. Expansion of these interventions to reach farmworkers, regardless of immigration or health insurance status, may need creative strategies, e.g., collaboration with *Promotoras*.

Furthermore, increasing access to affordable health care and expansion of eligibility for safety-net programs (i.e., Supplemental Nutrition Assistance Program (SNAP), called CalFresh in California) are policy changes that could protect or improve farmworkers' health. However, immigration status has been documented as a major barrier to SNAP participation (73), and anti-immigrant policy (i.e., the 2019 changes to public charge determination) has led to a decline in Medicaid enrollment among immigrants (74). Thus, any efforts to expand participation in publicly funded programs in the farmworker population would be more successful if implemented in collaboration with community-based organizations already serving this population to address fear, distrust, and language barriers.

In summary, the study findings highlight the elevated risk of chronic health conditions in Latino farmworkers, in particular for obesity, and among farmworkers who may lack access to health care, which represents a large proportion of this population. Recent studies of farmworker health using current guidelines are lacking, which limited our ability to compare the current study findings for the other health outcomes. Still, differences in chronic health risks by sex were observed, with differing patterns depending on the outcome, suggesting that clinical and public health responses might need to be sex-specific. Further research is needed to disentangle the effects of longer residence/acculturation on chronic health outcomes among farmworkers, which may vary by sex (40). Finally, the study findings supported the potentially positive impact of higher income on reducing one chronic health condition, high total cholesterol, likely through improved access to healthy foods.

## Data availability statement

The original contributions presented in the study are included in the article/supplementary material, further inquiries can be directed to the corresponding author.

## Ethics statement

The studies involving human participants were reviewed and approved by the Institutional Review Board of the University of California, Davis. The participants provided their written informed consent to participate in this study.

## Author contributions

SM conceived the research question, conducted the statistical analysis, and wrote the manuscript. CF contributed to interpretation of results and manuscript writing. AG-L supported data analysis and manuscript writing. MS was the principal investigator of the three studies included in this secondary data analysis, and critically reviewed the manuscript. All authors read and approve the manuscript before submission.

## Funding

Funding for the MICASA was provided by the National Institute for Occupational Safety and Health of the National Institutes of Health (Grant numbers 2U500H007550 and R01OH009293) and a grant from the California Endowment. The PASOS SALUDABLES Pilot Study was supported by a grant from Reiter Affiliated Companies, Oxnard, California, and the UC Davis Western Center for Agricultural Health and Safety (Grant number CDC-NIOSH U54 OH007550). The PASOS RCT study was supported by the National Institute of Diabetes and Digestive and Kidney Diseases of the National Institutes of Health (Grant number R18DK096429) and the UC Davis Western Center for Agricultural Health and Safety (Grant number CDC-NIOSH U54 OH007550). Publication made possible in part by support from the Berkeley Research Impact Initiative (BRII) sponsored by the UC Berkeley Library.

## Conflict of interest

The authors declare that the research was conducted in the absence of any commercial or financial relationships that could be construed as a potential conflict of interest.

## Publisher's note

All claims expressed in this article are solely those of the authors and do not necessarily represent those of their affiliated organizations, or those of the publisher, the editors and the reviewers. Any product that may be evaluated in this article, or claim that may be made by its manufacturer, is not guaranteed or endorsed by the publisher.
